# Parathyroid Hormone-Related Peptide and Its Analog, Abaloparatide, Attenuate Lethal Myocardial Ischemia-Reperfusion Injury

**DOI:** 10.3390/jcm11092273

**Published:** 2022-04-19

**Authors:** Joseph Wider, Vishnu V. R. Undyala, Beate Lanske, Nabanita S. Datta, Karin Przyklenk

**Affiliations:** 1 Department of Emergency Medicine, University of Michigan, Ann Arbor, MI 48109, USA; jwider@med.umich.edu; 2 Department of Physiology, Wayne State University School of Medicine, Detroit, MI 48201, USA; undya1v@cmich.edu; 3 Clinical Research Institute, Children’s Hospital of Michigan, Detroit, MI 48201, USA; 4 Department of Pediatrics, Central Michigan University, Detroit, MI 48201, USA; 5 Radius Health Inc., Boston, MA 02210, USA; blanske@radiuspharm.com; 6 Department of Internal Medicine, Wayne State University School of Medicine, Detroit, MI 48201, USA; ndatta@med.wayne.edu; 7 Department of Emergency Medicine, Wayne State University School of Medicine, Detroit, MI 48201, USA

**Keywords:** myocardial ischemia, myocardial infarction, infarct size, ischemia-reperfusion injury, cardioprotection, parathyroid hormone-related peptide, abaloparatide

## Abstract

Parathyroid hormone-related peptide (PTHrP) is well-known to play a role in bone formation, and abaloparatide, an analog of PTHrP(1-34), is approved for the treatment of osteoporosis in post-menopausal women. PTHrP has also been reported to have cardiovascular effects, with recent data demonstrating that exogenously administered PTHrP can limit the death of isolated cardiomyocytes subjected to oxidative stress via upregulation of classic ‘survival kinase’ signaling. Our aim in the current study was to extend this concept and, employing both in vitro and in vivo models, establish whether PTHrP(1-36) and abaloparatide are cardioprotective in the setting of lethal myocardial ischemia-reperfusion injury. We report that preischemic administration of PTHrP(1-36) and abaloparatide attenuated cell death in HL-1 cardiomyocytes subjected to simulated ischemia-reperfusion, an effect that was accompanied by the augmented expression of phospho-ERK and improved preservation of phospho-Akt, and blocked by co-administration of the MEK-ERK inhibitor PD98059. Moreover, using the translationally relevant swine model of acute coronary artery occlusion-reperfusion, we make the novel observation that myocardial infarct size was significantly reduced in pigs pretreated with PTHrP(1-36) when compared with placebo-controls (13.1 ± 3.3% versus 42.0 ± 6.6% of the area of at-risk myocardium, respectively; *p* < 0.01). Taken together, these data provide the first evidence in support of the concept that pretreatment with PTHrP(1-36) and abaloparatide renders cardiomyocytes resistant to lethal myocardial ischemia-reperfusion injury.

## 1. Introduction

Parathyroid hormone-related peptide (PTHrP) is a calcium-regulating peptide that was first identified in patients with malignancy-associated hypercalcemia [1]. PTHrP is well-recognized to play a role in bone formation [1,2,3] along with abaloparatide, an analog of PTHrP(1-34), approved in 2017 for the treatment of osteoporosis in post-menopausal women at high risk for bone fractures [4,5,6,7]. In addition, there is long-standing evidence that the physiologic effects of PTHrP extend beyond calcification and bone mineralization, i.e., PTHrP is constitutively expressed in multiple tissues and organs, including the heart and vascular smooth muscle, and reportedly contributes to the regulation of vascular tone and myocardial contractility [1,8,9].

PTHrP initiates its physiologic actions in target tissues by binding to the G protein-coupled type-1 parathyroid hormone receptor (PTH1R) [2,3]. The canonical intracellular signaling pathway triggered by PTHrP-PTH1R binding, first described in bone, is characterized by activation of both adenylyl cyclase/protein kinase A and phospholipase C, and culminates in the phosphorylation of multiple nuclear transcription factors [1,2,3,8,9]. More recently, a third subcellular signaling pathway has been described: although the molecular mechanisms are complex and incompletely resolved, PTH1R stimulation has been reported to increase the activation-phosphorylation of mitogen-activated protein kinases (MAPKs), an effect that is achieved via inhibition of mitogen-activated protein kinase phosphatase-1 (MKP-1) [1,10,11,12].

These latter data, together with evidence that PTHrP-PTH1R is expressed in the heart [1,8,9,13], raise the intriguing possibility that PTHrP may have as-yet unappreciated cardioprotective effects. Specifically, PTHrP may act as a preconditioning-mimetic agent (i.e., may limit ischemia-reperfusion-induced cardiomyocyte death and reduce myocardial infarct size) [1], potentially via activation of one or more ‘survival kinases’ that have been identified to contribute to the infarct-sparing effect of ischemic conditioning [14,15,16,17,18,19]. Support for the concept of PTHrP-induced cardioprotection was provided by results from our group demonstrating that pretreatment with PTHrP(1-36) attenuated the death of primary adult mouse cardiomyocytes exposed to oxidative stress, with upregulation of ‘survival kinases’ (extracellular signal-regulated kinase (ERK) ½ and phosphatidylinositol 3 (PI3) kinase/Akt) implicated to play a mechanistic role [20]. However, the cardioprotective efficacy of PTHrP has, to date, not been investigated in the setting of lethal ischemia-reperfusion injury. Accordingly, using both in vitro and in vivo models, our objective was to establish whether pretreatment with PTHrP(1-36) mitigates ischemia-reperfusion-induced cardiomyocyte death.

## 2. Materials and Methods

### 2.1. PTHrP(1-36) and Abaloparatide Assessed In Vitro

Protocols 1 and 2 were conducted using immortal HL-1 cardiomyocytes as described previously by our group [21,22]. HL-1 cardiomyocytes are a standard and accepted cell line in cardiovascular research. This model has been utilized in >1100 PubMed publications, including studies that have focused on cell death in the setting of (simulated) ischemia-reperfusion [23,24]. Before initiating experiments, HL-1 cells were grown to ~80% confluence in 95% room air/5% CO_2_ at 37 °C in Claycomb medium supplemented with fetal bovine serum (10%), L-glutamine (2 mM), norepinephrine (0.1 mM), penicillin (100 U/mL) and streptomycin (100 µg/mL) [21,22]. The medium and all components were purchased from Sigma Aldrich, St. Louis, MO, USA.

#### 2.1.1. Protocol 1: Effect of PTHrP(1-36) and Abaloparatide on HL-1 Cell Viability

The goal of Protocol 1 was to assess the effect of PTHrP(1-36) and abaloparatide on cell viability. In **Protocol 1A**, HL-1 cells were incubated for 24 h with (Tyr^36^)-PTH-Related Protein (1-36) (PTHrP(1-36): 10 nM, 100 nM; Bachem Inc., Torrance, CA, USA, Catalog # H-3208) or volume-matched vehicle (medium alone: *n* = 4 independent replicates per group). At 24 h after the onset of treatment, cells were subjected to 2 h of simulated ischemia (SI) + 24 h reoxygenation. During SI, Claycomb medium containing drug/placebo was replaced with drug-free hypoxia buffer at pH 6.6 (composed of NaCl (125 nM), KCl (8 mM), KH_2_PO_4_ (1.2 mM),1.25 mM MgSO_4_ (1.25 mM), CaCl_2_ (1.2 mM), NaHCO_3_ (6.25 mM), HEPES (20 mM), glucose (5.5 mM), 2-deoxy-d-glucose (20 mM), Na-lactate (5 mM): all ingredients from Sigma Aldrich, St. Louis, MO, USA) and the culture plates were sealed in a hermetic chamber with GasPak EZ Gas Generating Sachets (BD Biosciences, San Jose, CA, USA). Reoxygenation was achieved by removing the plates from the sealed chamber and exchanging the hypoxia buffer with serum-free Claycomb medium [21,22]. Additional time- and treatment-matched normoxic cultures served as controls (*n* = 4 independent replicates per group).

The design of **Protocol 1B** was identical, except cells were pretreated for 24 h with abaloparatide (0.1 nM, 1.0 nM, 10 nM, 100 nM: provided by Radius Health Inc., Boston, MA, USA) or volume-matched vehicle (medium alone) before being subjected to 2 h SI + 24 h reoxygenation or a matched normoxic period (*n* = 5 independent replicates per group). In **Protocol 1C**, a concurrent assessment of PTHrP(1-36) (10 nM, 100 nM) and abaloparatide (10 nM, 100 nM) was performed (*n* = 3 independent replicates per group).

The primary endpoint for Protocols 1A–C was HL-1 cell viability assessed using the MTT (3-(4,5-dimethythiazol-2-yl)-2,5-diphenyl tetrazolium bromide) assay (Sigma Aldrich, St. Louis, MO, USA). This well-established colorimetric method is based on the reduction of the soluble tetrazolium salt to purple formazan crystals by viable, metabolically active cells and quantified by measuring absorbance at 570 nm [25].

#### 2.1.2. Protocol 2: Role of Survival Kinases

Previous studies from our group revealed that pretreatment of isolated murine cardiomyocytes with PTHrP(1-36) was associated with increased phosphorylation-activation of ERK and Akt following exposure to oxidative stress [20]. Our aim in Protocol 2 was to extend this concept and obtain insight into the effect of PTHrP(1-36) and abaloparatide on the expression of phospho-ERK and phospho-Akt in HL-1 cardiomyocytes subjected to simulated ischemia-reperfusion.

In **Protocol 2A**, HL-1 cells, grown to ~80% confluence, were incubated for 24 h with PTHrP(1-36) (10 nM), abaloparatide (10 nM) or a vehicle as described in Protocol 1. At 24 h after the onset of treatment, cells underwent 2 h of SI + 10 min reoxygenation, while additional time- and treatment-matched normoxic cultures served as controls (*n* = 3 independent replicates per group).

At 10 min post-reoxygenation, cells were harvested and lysed in RIPA buffer containing 2 protease and phosphatase inhibitors. The lysates were centrifuged at 14,000× *g* for 15 min, and the resultant supernatant was collected and probed for the expression of ERK (total and phosphorylated: antibodies from Cell Signaling Technology Inc., Danvers, MA, USA), PI3 kinase/Akt (total and phosphorylated: Cell Signaling Technology Inc., Danvers, MA, USA) and GAPDH (Sigma Aldrich Inc., St. Louis, MO, USA) using standard methods [20,21,26]. Immunoreactive bands were identified by incubation with horseradish peroxidase (HRP)-conjugated secondary antibody, visualized using x-ray film, and quantified using the NIH ImageJ software.

In **Protocol 2B**, HL-1 cardiomyocytes were incubated for 24 h with PTHrP(1-36) (10 nM), abaloparatide (10 nM) or a vehicle, with or without the addition of PD98059 (pharmacologic inhibitor of MEK-ERK: 5 µM, Cell Signaling Technology Inc., Danvers, MA, USA). At 24 h after the onset of treatment, cells underwent either 2 h of SI + 24 h reoxygenation or a time-matched period of normoxia (*n* = 2 independent replicates per group). At 24 h following relief of SI, cell viability was quantified using the MTT assay as described in Protocol 1.

### 2.2. PTHrP(1-36) Assessed In Vivo

Protocols 3 and 4 focused on investigating the cardioprotective efficacy of the parent peptide, PTHrP(1-36), and were conducted using the swine model of coronary artery occlusion-reperfusion [27]. This preclinical model of acute myocardial infarction was chosen based on its established translational relevance, i.e., the pig and human heart are anatomically similar, and the temporal and spatial development of cardiomyocyte necrosis is comparable in pig and human myocardia [23]. All experiments were approved by the Institutional Animal Care and Use Committee of Wayne State University and were conducted in accordance with the *Guide for the Care and Use of Laboratory Animals: Eighth Edition*. Washington, DC: The National Academies Press, https://doi.org/10.17226/12910.

#### 2.2.1. Protocol 3: Pharmacokinetics of PTHrP(1-36) Following Administration of Peptide

The aim of Protocol 3 was to determine the temporal profile of PTHrP(1-36) in circulating blood following administration of the peptide. Female domestic swine (*n* = 3; body weight = ~30 kg, purchased from the Michigan State University Swine Farm) were pretreated with carprofen (4.4 mg/kg PO) and anesthetized with an intramuscular injection of midazolam (0.4 mg/kg) + xylazine (1.0 mg/kg) + butorphanol (0.2 mg/kg). The pigs were then intubated and ventilated with room air, with anesthesia maintained by the inhalation of isoflurane (1.5–2%). Under sterile conditions, a surgical cut-down was performed and a cannula was inserted in the right jugular vein. After obtaining an initial baseline blood sample (volume: 3 mL), 15 µg PTHrP(1-36) was dissolved in 500 µL sterile saline and administered by subcutaneous injection (the route used for clinical administration of abaloparatide) [4,5,6]. Subsequent serial blood samples were obtained at 5, 10, 15, 30, 45, 60, 90 and 120 min post-treatment. All samples were collected in vacutainers containing EDTA as the anticoagulant, and aprotinin (100 µL per mL of blood) was added to each sample immediately after collection. Samples were centrifuged for 12 min at 1500× *g* and the plasma was collected and stored at −80 °C until analyzed. After obtaining the final blood sample, the jugular cannula was removed, the incision was sutured, and anesthesia was discontinued. Animals were returned to their cages, allowed to recover for 1–2 weeks, and enrolled in Protocol 4.

The primary endpoint, plasma PTHrP concentration (in ng/mL) was quantified using a standard commercial ELISA kit as per the manufacturer’s instructions (Phoenix Pharmaceuticals Inc., Burlingame, CA, USA, Catalog # EK-056-04), with all samples assayed in duplicate.

#### 2.2.2. Protocol 4: Effect of PTHrP(1-36) on Infarct Size

The objective of Protocol 4 was to establish whether pretreatment with PTHrP(1-36) renders the in vivo swine heart resistant to lethal ischemia-reperfusion injury and reduces myocardial infarct size using standard methods described previously [27]. A total of 15 female domestic swine (weight: ~30–35 kg, purchased from the Michigan State University Swine Farm) were enrolled, including animals previously utilized in Protocol I (*n* = 3) and 12 additional naïve animals. The target enrollment was based on previous experience from our group and others with the swine model, together with power analysis. Specifically, we anticipated that infarct size in the placebo-control group would average approximately 45% of the risk region, and hypothesized that pretreatment with PTHrP would be cardioprotective and evoke a ~40–50% reduction of infarct size. For this predicted difference to achieve significance by unpaired *t*-test, given the typical variance in the data, power analysis (with α = 0.05 and β = 0.10) indicated that approximately 6–8 successful experiments will be required in each treatment group. In an effort to ensure the rigor of the study, the protocol was randomized, PTHrP(1-36)/vehicles were administered in a blinded manner, the surgical preparation was performed by a member of the investigative team who was unaware of the group assignment, and the data were analyzed without knowledge of the treatment group.

Pigs were assigned to receive PTHrP(1-36) (1 µg/kg in 500 µL sterile saline) or a vehicle (sterile saline alone). PTHrP(1-36) or placebo was administered once per day for 3 days via subcutaneous injection, with a 4th dose administered ~5 min before the onset of coronary artery occlusion.

All animals were anesthetized as described in Protocol 3. Core temperature was maintained at 38 °C using a Blanketrol^®^ heating pad (Gentherm, Cincinnati, OH, USA), and hemodynamics, spO_2_ and end-expiratory CO_2_ were monitored throughout each experiment (SurgiVet^®^: Smiths Medical Inc., Minneapolis, MN, USA). The left jugular vein was cannulated for administration of fluids, the heart was exposed via a midline sternotomy, and a segment of the left anterior descending coronary artery (LAD) was isolated, typically midway along its course and distal to the first major diagonal branch.

After stabilization, pigs received the final dose of PTHrP(1-36) or a vehicle and, 5 min later, the LAD was occluded by placing an atraumatic vascular clamp on the isolated arterial segment. At 10–12 min post-occlusion, lidocaine (1.3 mg/kg) was administered via the jugular vein in an effort to minimize the incidence of lethal ventricular fibrillation (VF). At 75 min post-occlusion, pigs received an additional dose of lidocaine, and the LAD was reperfused by removal of the vascular clamp. For animals that developed VF at any time during the protocol, resuscitation was attempted by applying DC countershocks directly to the heart (energy of 20–50 Joules; maximum of 4 attempts).

At 3 h post-reperfusion, the LAD was ligated at the site of the previous occlusion and Unisperse blue pigment (Ciba Specialty Chemicals Corp., Tarrytown, NY, USA) was injected IV to delineate the area at risk (AR), or volume of myocardium rendered ischemic during coronary occlusion. Pigs were then euthanized under deep anesthesia by IV injection of Fatal-Plus^®^ (Vortech Pharmaceuticals Ltd., Southfield, MI, USA). The hearts were rapidly excised, cut into 5–6 transverse slices, and photographed. The heart slices were immediately incubated in triphenyltetrazolium chloride (Sigma Aldrich, St. Louis, MO, USA; 10 min at 37 °C) to discern infarcted versus viable tissue and re-photographed.

The primary endpoint of Protocol 4 was the quantitative assessment of myocardial infarct size. The area at risk (AR) and area of necrosis (AN) in each heart slice were measured from the photographs using ImageJ, corrected for tissue weight, and summed for each heart. AR was expressed as a % of the total left ventricular (LV) weight, and AN was expressed as a % of the AR [27]. In addition, heart rate and arterial pressure were and tabulated at baseline and at 15 min, 1 h and 3 h post-reperfusion, and the incidence of VF was recorded for each pig.

#### 2.2.3. Statistical Analysis

All data were analyzed using Prism version 9 (GraphPad Software, San Diego, CA, USA). For Protocols 1 and 2A, HL-1 cell viability and kinase expression were compared among groups using analysis of variance (ANOVA) and, if significance was achieved, pairwise comparisons were made using Tukey’s test. For Protocol 2B, statistical comparisons were not made because of the small number of replicates performed, while Protocol 3 was descriptive. For Protocol 4, AN/AR and AR/LV were compared between control and PTHrP(1-36)-treated groups by unpaired two-tailed Student’s *t*-tests, the incidence of VF was compared using Fisher’s exact test, and hemodynamic endpoints were compared using 2-factor ANOVA (for group and time) with replication. Results are reported as mean ± SEM, and *p*-values < 0.05 were considered statistically significant.

## 3. Results

### 3.1. PTHrP(1-36) and Abaloparatide Assessed In Vitro

#### 3.1.1. Protocol 1: Effect of PTHrP(1-36) and Abaloparatide on HL-1 Cell Viability

**Figure 1:**
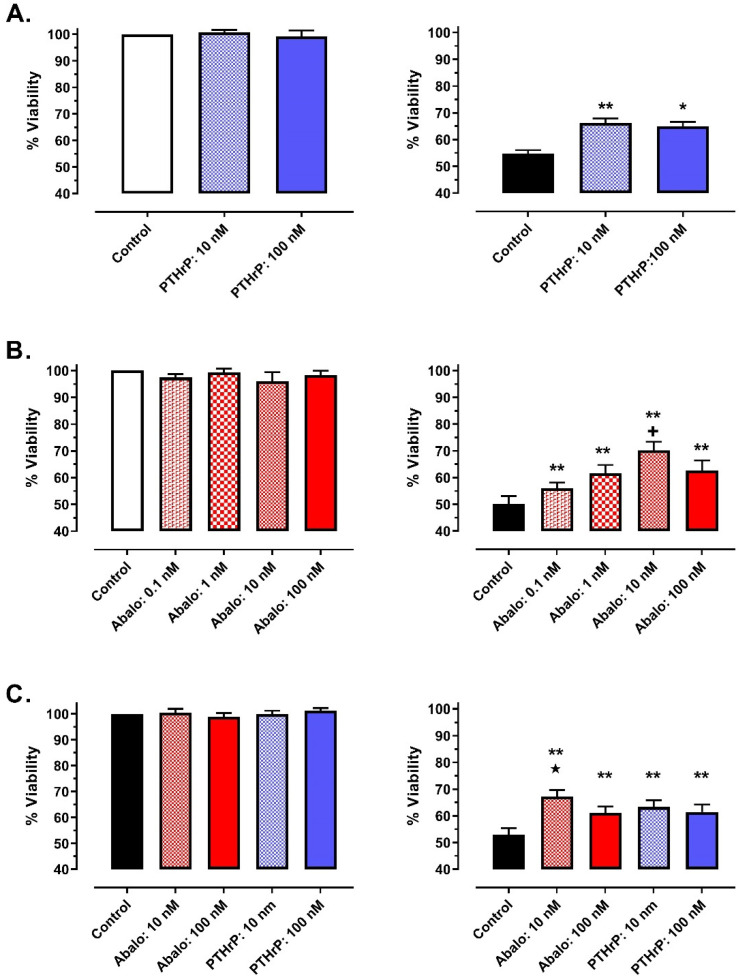
Effect of PTHrP(1-36) (PTHrP: **Panel A**), abaloparatide (Abalo: **Panel B**) and concurrent assessment of PTHrP(1-36) and Abalo (**Panel C**) on viability of HL-1 cardiomyocytes maintained under normoxic conditions (**left panels**) or subjected to simulated ischemia-reperfusion (**right panels**). Data reported as mean ± SEM. * *p* < 0.05, ** *p* < 0.01 versus matched vehicle-control group; + *p* < 0.05 versus Abalo 0.1 nM, 1 nM and 100 nm; ★ *p* < 0.05 versus Abalo 100 nM, PTHrP 10 nM and PTHrP 100 nM.

In HL-1 cardiomyocytes subjected to SI-reoxygenation, cell viability was significantly better maintained in cells pretreated with PTHrP(1-36) or abaloparatide (Figure 1). For PTHrP, % viability was comparable with both 10 nM and 100 nM of the peptide (Protocol 1A, Figure 1A—right panel) while, for abaloparatide (Protocol 1B, Figure 1B—right panel), the magnitude of protection was modestly but significantly greater in cells incubated with 10 nM of the agent. Concurrent evaluation of both agents (Protocol 1C) confirmed these observations (Figure 1C—right panel). In contrast, under normoxic conditions, incubation with PTHrP(1-36) or abaloparatide had no effect on HL-1 cell viability (Figure 1—left panels).

#### 3.1.2. Protocol 2: Role of Survival Kinases

**Figure 2:**
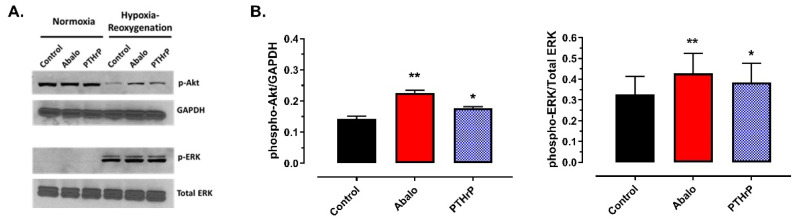
Effect of PTHrP(1-36) (PTHrP) and abaloparatide (Abalo) on phospho-Akt and phospho-ERK expression in HL-1 cells subjected to simulated ischemia-reperfusion. (**A**) original immunoblots; (**B**) Mean phospho-Akt expression (normalized to GAPDH), mean phospho-ERK expression (normalized to total ERK) ± SEM. * *p* < 0.05, ** *p* < 0.01 versus control group.

Under normoxic conditions, all Akt was present in its phosphorylated form (not shown), while all ERK was unphosphorylated, with no differences among groups (Protocol 2A, Figure 2A). Accordingly, for quantitation, expression of phospho-Akt was normalized to GAPDH, and expression of phospho-ERK was normalized to total ERK.

In vehicle-treated cells subjected to SI-reoxygenation, expression of phospho-Akt/GAPDH averaged 0.14 ± 0.01, while the mean expression of phospho/total ERK was 0.33 ± 0.09. Pretreatment with PTHrP(1-36) and abaloparatide was associated with better preservation of phospho-Akt and a modest but significant increase in phospho-ERK expression (Protocol 2A, Figure 2B).

**Figure 3:**
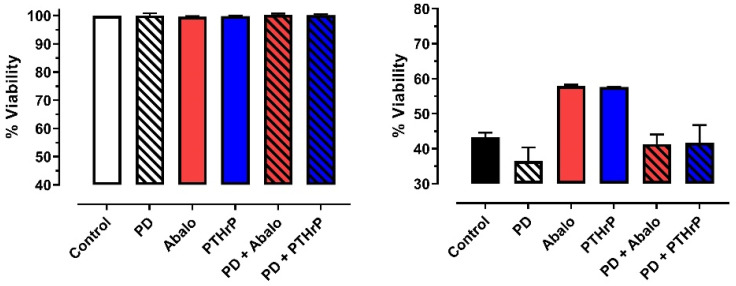
Effect of vehicle, PTHrP(1-36) (PTHrP), abaloparatide (Abalo), PD998059 (PD) + vehicle, PD98059 + PTHrP or PD98059 + Abalo on viability of HL-1 cardiomyocytes maintained under normoxic conditions (**left panel**) or subjected to simulated ischemia-reperfusion (**right panel**). Data reported as mean ± SEM.

In Protocol 2B: under normoxic conditions, neither PTHrP(1-36), abaloparatide, or PD98059, given alone or in combination, had an effect on HL-1 cell viability (Figure 3—left panel). As expected from Protocol 1, pretreatment with either PTHrP(1-36) or abaloparatide attenuated SI-reoxygenation-induced cell death when compared with the vehicle-treated cohort. The protective effect of PTHrP(1-36) and abaloparatide were, however, abrogated by co-administration of PD98059 (Figure 3—right panel)

### 3.2. PTHrP(1-36) Assessed In Vivo

#### 3.2.1. Protocol 3: Pharmacokinetics of PTHrP(1-36) Following Administration of Peptide

**Figure 4:**
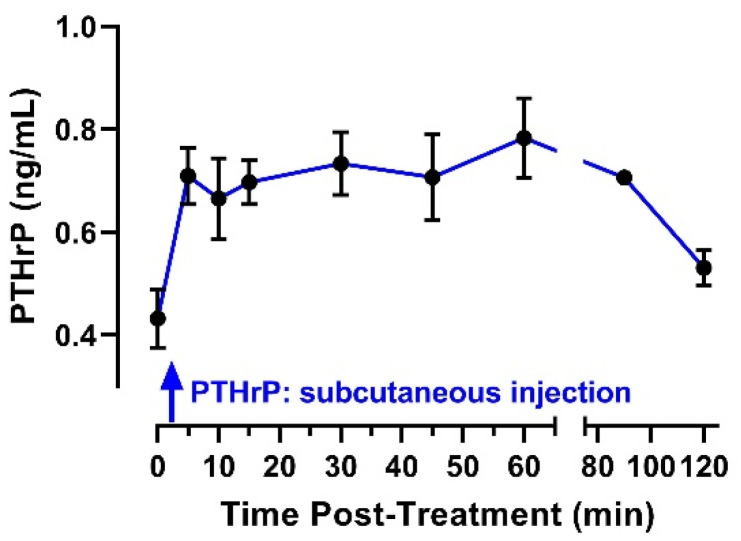
Plasma PTHrP concentration (ng/mL) in anesthetized pigs during the initial 2 h following subcutaneous injection. Data reported as mean ± SEM.

Endogenous plasma PTHrP concentrations measured at baseline, before administration of exogenous peptide, averaged 0.43 ng/mL (Figure 4). Plasma PTHrP concentration increased rapidly following injection (mean of 0.71 ng/mL at 5 min post-injection) and remained approximately constant throughout the initial 90 min post-treatment (Figure 4). This temporal profile suggests that, for experiments conducted in Protocol 4, PTHrP(1-36) concentrations were increased at the onset of coronary occlusion and remained elevated throughout ischemia and the initial minutes of reperfusion.

#### 3.2.2. Protocol 4: Effect of PTHrP(1-36) on Infarct Size

Of the 15 swine enrolled in the study, 8 were randomized to receive PTHrP and 7 were assigned to the vehicle-control cohort. As expected, myocardial ischemia-reperfusion in the pig is characterized by a high incidence of VF [23]: 12/15 animals developed one or more episodes of VF during LAD occlusion or immediately upon reperfusion (*n* = 7 in the PTHrP(1-36)-treated group; *n* = 5 in controls; *p* = 0.57 (ns)). Nine pigs were resuscitated by application of 1–3 25 J DC shocks directly to the heart: *n* = 5 that received PTHrP(1-36) and *n* = 4 controls. The remaining 3 animals that developed VF (1 control, 2 PTHrP-treated) failed to respond to defibrillation and died during LAD occlusion. Accordingly, 12 pigs successfully completed the protocol: *n* = 6 per group.

**Figure 5:**
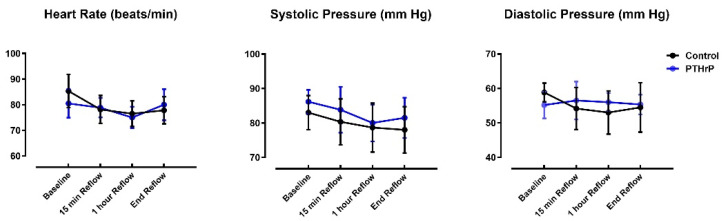
Heart rate, systolic and diastolic pressures, measured at baseline, 15 min, 1 h and 3 h (end) following reperfusion in PTHrP(1-36)-treated pigs and vehicle-controls. Data reported as mean ± SEM; no significant differences between groups.

**Figure 6:**
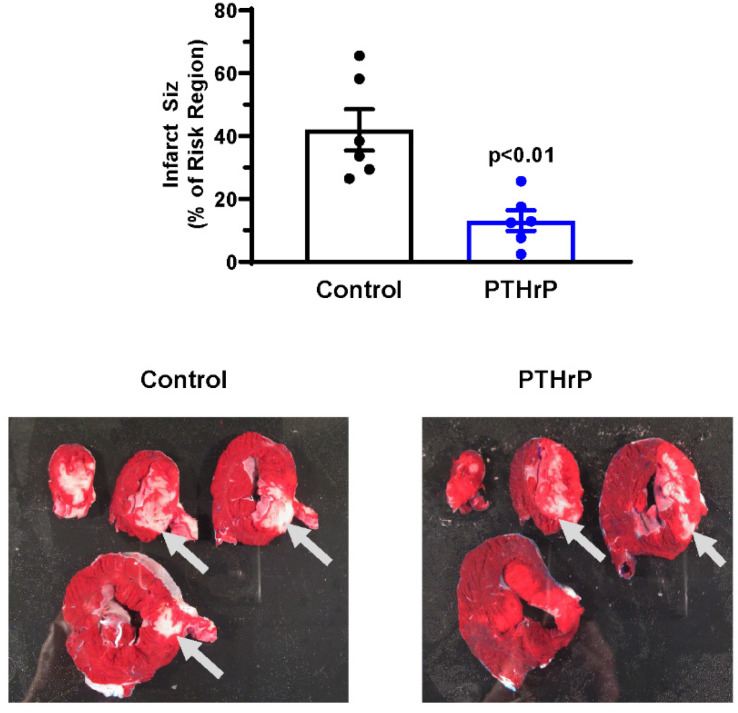
Myocardial infarct size in PTHrP(1-36)-treated pigs and vehicle-controls. (**Top panel**) individual data points and mean values ± SEM. (**Bottom panel**) Original images of heart slices from one control and one PTHrP(1-36)-treated pig. Heart slices are stained with triphenyltetrazolium chloride; using this method, regions of necrotic myocardium (highlighted by gray arrows) remain unstained and appear pale.

Heart rate, systolic and diastolic pressure were comparable in control and PTHrP(1-36)-treated pigs at baseline and throughout reperfusion (Figure 5). In addition, AR, expressed as a % of total LV weight did not differ between groups, averaging 16.9 ± 1.1% and 17.6 ± 1.0% in the control and PTHrP(1-36)-treated groups, respectively (*p* = 0.60 (ns); not shown). Mean infarct size in the control cohort was 42.0 ± 6.6% of the AR. In contrast, AN/AR was significantly reduced in pigs pretreated with PTHrP(1-36) at an average of 13.1 ± 3.3% (*p* < 0.01; Figure 6).

## 4. Discussion

In this study, we make the novel observation that pretreatment with PTHrP(1-36) and abaloparatide favorably attenuate lethal myocardial ischemia-reperfusion injury. Using the HL-1 cardiomyocyte model, we report that preischemic administration of PTHrP(1-36) and abaloparatide was associated with better maintenance of cell viability, augmented expression of phospho-ERK and improved preservation of phospho-Akt following simulated ischemia-reperfusion. Moreover, co-administration of PD98059 attenuated the cardioprotective effect of PTHrP(1-36) and abaloparatide, an outcome that is consistent with the concept that the better maintenance of HL-1 cell viability may be due, in part, to increased activation-phosphorylation of ERK. Finally, in the translationally relevant swine model, our randomized and blinded study reveals that myocardial infarct size was significantly reduced in pigs pretreated with PTHrP(1-36) when compared with placebo-controls.

### 4.1. Cardiovascular Effects of PTHrP

Data obtained largely from rodent models have demonstrated that PTHrP plays a physiologic role in the maintenance of vascular tone, control of coronary blood flow, and regulation of heart rate [8,9,28,29,30,31]. PTHrP has also been implicated to act as a positive inotrope [32]; however, it remains unclear whether this is an indirect consequence of the effect of the peptide on heart rate and blood flow or a direct effect on myocardial contractility *per se* [31,33]. In this regard, it is interesting to note that PTHrP had no apparent inotropic consequences when administered intravenously to human volunteers [34].

### 4.2. PTHrP in Models of Ischemia-Reperfusion

Current evidence, albeit limited, suggests that PTHrP may have cardiovascular effects that extend beyond the physiologic maintenance of vascular tone, chronotropy and inotropy. For example, increased expression and activity of PTHrP-PTH1R have been described in ischemic human ventricular cardiomyocytes and in cardiac samples obtained from patients with heart failure [13,35], and an augmented contractile response to exogenous administration of PTHrP has been reported in porcine myocardium (and isolated porcine cardiomyocytes) following relief of a brief ischemic episode [31]. In addition, 4 weeks of treatment with PTHrP (in this case, PTHrP(87-139), the nuclear localization sequence of the peptide) has recently been shown to attenuate molecular indices of apoptosis, stimulate angiogenesis and improve left ventricular function in a murine model of permanent coronary occlusion [36]. Most notably, among the small number of studies that have assessed the effects of PTHrP in preclinical models of ischemia-reperfusion, there is agreement that administration of the peptide confers a benefit [31,37,38]. However, the primary endpoint of these previous reports was the acute recovery of contractile function of viable but stunned post-ischemic myocardium rather than measurement of myocardial infarct size or cardiomyocyte viability (the hallmark and gold standard of cardioprotection [23]) in the setting of lethal ischemia-reperfusion injury.

Two published reports provide indirect support for the concept that PTHrP may render cardiomyocytes resistant to ischemia-reperfusion-induced cell death. Data from our group demonstrated that cell viability was better maintained in primary adult mouse cardiomyocytes pretreated with PTHrP(1-36) and exposed to oxidative stress [20], a well-established mechanistic component of ischemia-reperfusion injury [15,18,19,39,40]. Second, activation of the extracellular calcium-sensing receptor, which contributes to the regulation of PTHrP secretion, has been implicated to play a role in the infarct-sparing effect of ischemic preconditioning [41]. Results of the current study extend these observations and provide the first direct evidence that pretreatment with PTHrP(1-36) attenuates cardiomyocyte death and reduces infarct size.

Interestingly, in Protocol 4, we observed no differences in heart rate or arterial pressures between PTHrP(1-36)- and placebo-treated pigs. This is in apparent contrast to the hemodynamic effects of abaloparatide administered to post-menopausal women with osteoporosis: subcutaneous injection of the agent was associated with a modest and transient increases in heart rate and decreases in supine blood pressure [6]. The reasons for this difference are unknown, but in addition to potential species-specific effects, may reflect the influence of the isoflurane anesthesia used in our model. Most importantly, there was no evidence of an increase in the incidence of adverse cardiac events in subjects treated with the PTHrP analog [6].

### 4.3. Strengths, Limitations and Future Directions

Strengths of this study include the comprehensive assessment of PTHrP(1-36) (and abaloparatide) on cardiomyocyte fate in the HL-1 cell model, together with the randomized and blinded study design employed for the evaluation of myocardial infarct size in the translationally relevant swine model. In addition, the repeated daily subcutaneous administration of PTHrP(1-36) in Protocol 4 mimics the manner in which osteoporosis patients are treated with abaloparatide. If these data showing infarct size reduction with preischemic administration of PTHrP are substantiated in future studies, the peptide could potentially be administered in the setting of planned ischemic events such as coronary artery bypass surgery (~200,000 cases per year in the USA alone [42]). However, the choice of the dose and 4-day duration of treatment were empiric, and it is unknown whether the infarct-sparing effect of PTHrP(1-36) is maintained if treatment is given as a single dose and initiated at the time of reperfusion. This first proof-of concept study was acute, with infarct size assessed hours (rather than days) post-reperfusion, and only female pigs (rather than both sexes) were enrolled. This latter issue is potentially important given the reported differences in the expression and function of PTHrP in female versus male rat hearts [38]. Finally, although the rationale for the study was based on evidence from other models showing that PTHrP-PTH1R signaling inhibits MKP-1 and, as a consequence, activates survival kinases, the protocols were not designed to provide a robust assessment of the mechanisms responsible for the cardioprotection achieved with PTHrP(1-36). In order to provide definitive evidence in support of the future evaluation of PTHrP or abaloparatide as a clinically relevant cardioprotective agent, additional experiments are required in which the peptide is administered upon relief of ischemia, both sexes are enrolled, mechanistic endpoints are incorporated, and infarct size (together with indices of left ventricular function) is quantified days to weeks after reperfusion. If, under these rigorous conditions, the efficacy of PTHrP is maintained and an infarct-sparing effect of the agent is demonstrated, PTHrP may, in future, be evaluated as an adjuvant therapy, administered at the time of reperfusion, for the clinical treatment of lethal reperfusion injury.

## Data Availability

The data presented in this study are available on request from the corresponding author.
